# The Effect of Fat Intake with Increased Omega-6-to-Omega-3 Polyunsaturated Fatty Acid Ratio in Animal Models of Early and Late Alzheimer’s Disease-like Pathogenesis

**DOI:** 10.3390/ijms242317009

**Published:** 2023-11-30

**Authors:** Pablo Galeano, Marialuisa de Ceglia, Mauricio Mastrogiovanni, Lorenzo Campanelli, Dina Medina-Vera, Nicolás Campolo, Gisela V. Novack, Cristina Rosell-Valle, Juan Suárez, Adrián Aicardo, Karen Campuzano, Eduardo M. Castaño, Sonia Do Carmo, A. Claudio Cuello, Silvina Bartesaghi, Rafael Radi, Fernando Rodríguez de Fonseca, Laura Morelli

**Affiliations:** 1 Laboratory of Brain Aging and Neurodegeneration, Fundación Instituto Leloir, IIBBA-CONICET, Av. Patricias Argentinas 435, Ciudad Autónoma de Buenos Aires C1405BWE, Argentina; pgaleano@leloir.org.ar (P.G.); lcampanelli@leloir.org.ar (L.C.); gnovack@leloir.org.ar (G.V.N.); kcampuzano@leloir.org.ar (K.C.); ecastano@leloir.org.ar (E.M.C.); 2 Grupo de Neuropsicofarmacología, Unidad Clínica de Neurología, IBIMA y Plataforma BIONAND, Hospital Universitario Regional de Málaga, Av. Carlos Haya 82, 29010 Málaga, Spain; marialuisa.deceglia@ibima.eu (M.d.C.); dina.medina@ibima.eu (D.M.-V.); crosell@asphalion.com (C.R.-V.); 3 Departamento de Bioquímica, Facultad de Medicina, Universidad de la República, Av. Gral. Flores 2125, Montevideo 11800, Uruguay; maurimastro@fmed.edu.uy (M.M.); ncampolo@fmed.edu.uy (N.C.); aaicardo@fmed.edu.uy (A.A.); sbartesa@fmed.edu.uy (S.B.); rradi@fmed.edu.uy (R.R.); 4 Centro de Investigaciones Biomédicas, Facultad de Medicina, Universidad de la República, Av. Gral. Flores 2125, Montevideo 11800, Uruguay; 5 Instituto de Investigación Biomédica de Málaga (IBIMA), Departamento de Anatomía Humana, Medicina Legal e Historia de la Ciencia, Universidad de Málaga, Bulevar Louis Pasteur 32, 29071 Málaga, Spain; juan.suarez@uma.es; 6 Departamento de Nutrición Clínica, Escuela de Nutrición, Universidad de la República, Av. Ricaldoni S/N, Montevideo 11600, Uruguay; 7 Department of Pharmacology and Therapeutics, McGill University, McIntyre Medical Building 3655 Prom. Sir-William-Osler, Montreal, QC H3G 1Y6, Canada; sonia.docarmo@mcgill.ca (S.D.C.); claudio.cuello@mcgill.ca (A.C.C.)

**Keywords:** nutrition, high fat diet, PUFA, omega-3/omega-6, Alzheimer’s disease, transgenic animals, neuropathology, oxidative stress, inflammation

## Abstract

This work aims to clarify the effect of dietary polyunsaturated fatty acid (PUFA) intake on the adult brain affected by amyloid pathology. McGill-R-Thy1-APP transgenic (Tg) rat and 5xFAD Tg mouse models that represent earlier or later disease stages were employed. The animals were exposed to a control diet (CD) or an HFD based on corn oil, from young (rats) or adult (mice) ages for 24 or 10 weeks, respectively. In rats and mice, the HFD impaired reference memory in wild-type (WT) animals but did not worsen it in Tg, did not cause obesity, and did not increase triglycerides or glucose levels. Conversely, the HFD promoted stronger microglial activation in Tg vs. WT rats but had no effect on cerebral amyloid deposition. IFN-γ, IL-1β, and IL-6 plasma levels were increased in Tg rats, regardless of diet, while CXCL1 chemokine levels were increased in HFD-fed mice, regardless of genotype. Hippocampal 3-nitrotyrosine levels tended to increase in HFD-fed Tg rats but not in mice. Overall, an HFD with an elevated omega-6-to-omega-3 ratio as compared to the CD (25:1 vs. 8.4:1) did not aggravate the outcome of AD regardless of the stage of amyloid pathology, suggesting that many neurobiological processes relevant to AD are not directly dependent on PUFA intake.

## 1. Introduction

The late-onset Alzheimer’s disease (AD), in which symptoms typically arise after age 65, is the main cause of dementia in the aging population [1]. Financial resources worldwide have been allocated in the last 20 years to develop effective pharmacological therapies to avoid or delay neuropathology and cognitive impairment in AD, but to date, none has been fully effective. Therefore, efforts currently focus on defining and testing non-pharmacological therapies to delay cognitive decline in AD. In this context, studies aimed to evaluate the impact of “modifiable” risk factors on AD are fundamental due to their potential effect on disease prevention at the population level. The strongest experimental evidence suggests that the non-genetic risk factors primarily associated with dementia are (1) aging; (2) low educational levels in childhood; (3) hypertension in middle age; and (4) smoking and type 2 diabetes mellitus (DM2) throughout life. Previous studies in humans and in animal models have shown that consuming energy-dense diets (high in saturated fat and refined sugar) is not only associated with weight gain and metabolic syndrome but also with hippocampal-dependent memory deficiencies and the development of hippocampal pathologies [2,3,4].

The hippocampus is the brain region that displays the main neuropathological characteristics of AD, including extracellular oligomeric amyloid β (Aβ) peptide and parenchymal amyloid plaque deposition and the intracellular accumulation of hyperphosphorylated tau protein (p-Tau), which are associated with synaptic dysfunction, neuroinflammation, and progressive cognitive deterioration [5,6].

According to the World Health Organization (WHO), sporadic AD is one of the so-called “diseases of civilization” because the onset and evolution of dementia are highly influenced by lifestyle factors, including diet. In this regard, in 2019, the WHO recommended focusing on modifiable risk factors to support future prevention strategies for AD. Apparently, adherence to the Mediterranean diet (characterized by a high intake of vegetables; fruits; legumes; fish; cereals; and unsaturated fat, mainly olive oil) combined with a diet focused on curbing hypertension was associated with better performance in terms of memory and therefore seem to be beneficial for preventing the development of AD. By contrast, substantial evidence suggests that an excess of saturated fatty acids and simple sugars in the diet could be an environmental risk factor for AD. On this point, animal models of AD allowed researchers to better investigate the causative mechanisms linking AD with diet. It was reported that an energy-dense diet in rodents (mice and rats) induces systemic alterations, including metabolic changes, obesity, adipose tissue inflammation, gut microbiota dysbiosis, the acceleration of systemic low-grade inflammation, the impairment of the blood–brain barrier, and the development of neuroinflammation and nitro-oxidative stress [7]. It was proposed that neuroinflammation-mediated nitro-oxidative stress is followed by the dysfunction of synaptic transmission, neurodegeneration, and finally memory and cognitive impairment.

Nitro-oxidative stress is broadly defined as stress initiated by the nitric oxide/superoxide/peroxynitrite (**^•^**NO/O_2_^−^**^•^**/ONOO^−^) cascade [8,9]. It has been recognized that ONOO^−^ may cause the nitration of tyrosine residues on proteins promoting the inactivation of several enzymes, including manganese superoxide dismutase (Mn-SOD) and aconitase affecting redox balance and mitochondrial respiratory chain functionality [10,11]. Notably, one of the brain enzymes mainly affected by nitro-oxidative stress is astrocytic glutamine synthetase (GS), which participates in the conversion of glutamate (released in the post-synapse and taken up by astrocytes) to glutamine (the energy substrate used by neurons). We have recently published the multiple oxidative GS modifications mediated by peroxynitrite, which lead to the inactivation and aggregation of the protein [12]. The low levels and reduced activity of GS have also been reported in the brains of patients with AD [13]. In the context of AD neuropathology, Aβ-induced **^•^**NO production from neuronal and brain microvasculature endothelial cells has been reported. In addition, high levels of endothelial nitric oxide synthase (eNOS) inhibitor asymmetric dimethylarginine (ADMA) in plasma have been linked to an increased risk for endothelial dysfunction associated with cognitive decline [14,15]. Also, Aβ increased the concentration of 3-nitrotyrosine (NO_2_-Tyr), a marker of ONOO^−^ formation [15]. However, studies linking the expression of nitro-oxidative stress biomarkers (NO_2_-Tyr; ADMA, and GS activity) with the type of diet are scarce.

The recommendation to eat less fat as a prophylactic approach for AD was challenged by the result of a meta-analysis of several well-controlled studies regarding AD according to which cognitively normal subjects showed no association between dietary fat consumption and the development of AD [16]. Moreover, several preclinical studies carried out in transgenic animal models of AD showed no consistent association between high-fat diet (HFD) consumption and the worsening of AD-like neuropathology (amyloid deposition and neuroinflammation) in part, probably, due to technical discrepancies in the transgenic AD models used, specific components of the diet, or the time course of HFD administration [17].

Recent statistics analysis showed that despite eating less fat, Americans gained weight at an alarming rate and displayed more cardiovascular disease [18]. This is why American guidelines now suggest that Americans limit added sugars and the consumption of sugary drinks. Therefore, the previous recommendation about limiting total fat intake was removed. Linoleic acid and α-linolenic acid are precursors of the omega-6 and omega-3 polyunsaturated fatty acid (PUFA) family. Experimental studies have suggested that omega-6 is primarily proinflammatory, while omega-3 has anti-inflammatory effects in AD [19]. Consequently, in this report, we aimed to shed light on whether the intake of an HFD with an elevated omega-6-to-omega-3 ratio as compared to the CD (25:1 vs. 8.4:1) negatively impacts the behavioral and neuropathological characteristics of AD. We analyzed the effect of a PUFA-enriched HFD (40.1% energy from fat based on corn oil) on two preclinical transgenic (Tg) models of AD-like amyloid pathology: the homozygous McGill-R-Thy1-APP rat [20] and the homozygous 5xFAD (C57BL6) mouse [21].

It is worth noting that while in the initial characterization of the model published by Leon et al. (2010) [20], the first plaques could be detected in the subiculum of homozygous McGill-R-Thy1-APP rats from 6 to 9 months of age, this phenotype has drifted since then. This is an occurrence that is frequent in most transgenic models. In recent reports, it has been documented that about 50% of homozygous McGill-R-Thy1-APP rats display plaques at 12 months of age [22,23,24]. In addition, at 6-month-old, homozygous McGill-R-Thy1-APP rats show increased intermediate activated microglia in the subiculum, and by 13 months of age, the increment in activated microglia is observed throughout the cortex and the hippocampal formation [25]. Moreover, at 18 months of age it has been observed a 22% reduction of neurons in the subiculum and by 20 months of age, a reduced number of cholinergic synaptic boutons was found. Cognitive deficits appear as early as 3 months of age and worsen with age [20]. On the other hand, 5xFAD (C57BL6) mice exhibit massive amyloid plaques at 2 months old in the hippocampus, the cortex, the thalamus, and the spinal cord associated with microgliosis, astrogliosis, and synapse degeneration; microgliosis is associated with vascular damage. A 40% neuronal loss was observed in cortical layer V at one year. Myelin abnormalities are present in mice as young as one month of age and become more severe with age. Age-dependent memory deficits, motor phenotype, and reduced anxiety emerge between 3 and 6 months of age and worsen with age (https://www.alzforum.org/research-models/alzheimers-disease. Accessed on 25 October 2023).

Taking into account the features of amyloid pathology and cognitive performance in each model, McGill-R-Thy1-APP Tg rats would represent earlier stages of the disease, while 5xFAD Tg mice represent more advanced ones. We exposed young rats (2 months old) and adult mice (3 months old) to a well-matched control diet (CD) and an HFD based on corn oil for 6 and 2.5 months, respectively. At the end of the nutritional paradigm, we carried out behavioral tests and recovered plasma and brain samples to assess the biomarkers of metabolic syndrome, neuropathology, inflammation, and protein nitration.

Our results show that an HFD based on polyunsaturated fatty acids (PUFAs) has limited deleterious effects on the outcome of AD, regardless of the stage of amyloid pathology.

## 2. Results

### 2.1. General Study Observations and Body Weight

**Figure 1:**
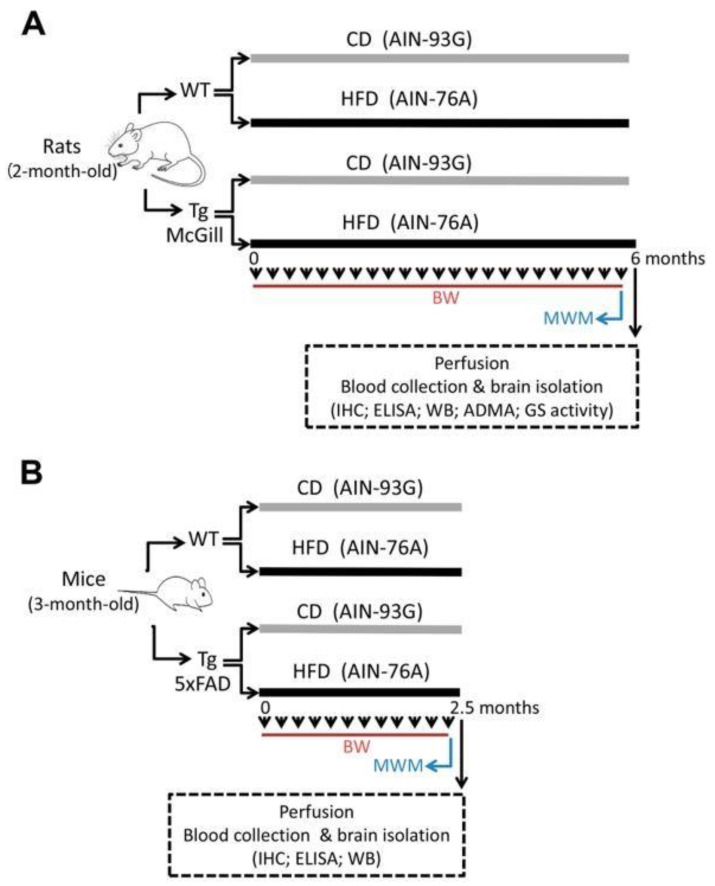
Experimental design: (**A**) two-month-old rats (30 WT and 30 Tg-McGill) and (**B**) three-month-old mice (10 WT and 11 Tg-5xFAD) were exposed to a control diet (CD; n = 15 WT rats; n = 15 Tg rats; n = 5 WT mice; n = 5 Tg mice) or a high-fat diet (HFD; n = 15 WT rats; n = 15 Tg rats; n = 5 WT mice; n = 6 Tg mice) for 6 and 2.5 months, respectively. Animals’ body weight (BW) was recorded weekly (arrowheads). The Morris water maze (MWM) behavioral test was performed at the end of the nutritional protocol. After that, animals were perfused, blood was collected, and the brain was isolated to perform immunohistochemistry (IHC), ELISA, Western blotting (WB), asymmetric dimethylarginine (ADMA) quantification, and an analysis of glutamine synthetase (GS) activity.

**Figure 2:**
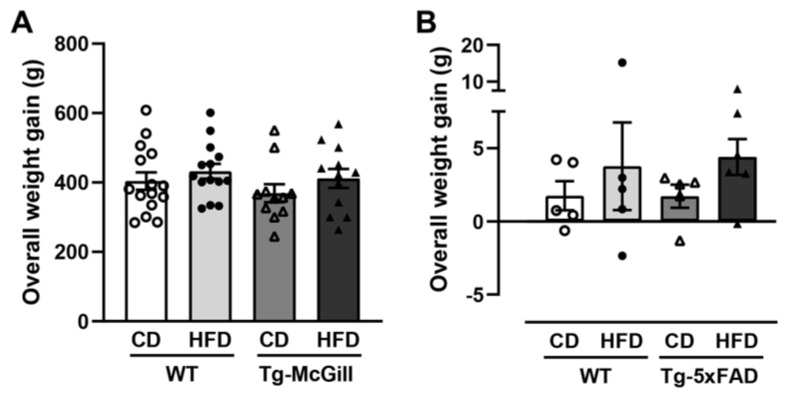
Overall weight gain of the animals that survive at the end of the nutritional paradigm. At the end of the experiment, the overall weight gain (g) (calculated as the final body weight-initial body weight) was not different between groups either in (**A**) rats (two-way ANOVA test: Genotype: F = 1.22, *p* = n.s.; Diet: F = 1.98, *p* = n.s.; Genotype × Diet: F < 1, *p* = n.s.) or in (**B**) mice (two-way ANOVA test: Genotype: F < 1, *p* = n.s.; Diet: F = 1.91, *p* = n.s.; Genotype × Diet: F < 1, *p* = n.s.). Data are shown as the mean ± SEM with individual values superimposed. N = 11-15 rats/group and N = 5–6 mice/group. WT, wild-type rats or mice; Tg-McGill, McGill-R-Thy1-APP transgenic rats; Tg-5xFAD, 5xFAD transgenic mice; CD, control diet; HFD, high-fat diet.

Nutritional paradigm started at 2 months of age for rats and 3 months of age for mice and finished after behavioral evaluation at 8 months of age for rats and 5.5 months of age for mice. A schematic representation of the experimental design is depicted in Figure 1. Mortalities occurred during the study in rats but not in mice. At the initiation of the study, transgenic (Tg) and wild-type (WT) rats (30 animals/genotype) and mice (10–11 animals/genotype) were randomly assigned to two test diet groups (HFD and CD). By the end of the experiment, the final number of animals was as follows: 15 WT-CD rats; 14 WT-HFD rats; 11 Tg-McGill-CD rats; 12 Tg-McGill-HFD; 5 WT-CD mice; 5 WT-HFD mice; 5 Tg-5xFAD-CD mice; and 6 Tg-5xFAD-HFD mice. At the start of the experiment, the body weights of Tg rats and mice were slightly lower than those of background strains, but in the case of Tg rats, this difference reached statistical significance (WT-rats: 360.0 g ± 8.5 g vs. Tg-McGill-rats: 278.3 g ± 14.9 g, *t* = 4.75, *p* < 0.0001; WT mice: 20.1 g ± 1.3 g vs. Tg-5xFAD mice: 18.5 g ± 0.9 g, *t* = 1.03, *p* = n.s.). Once experiments finished, the overall weight gain of the HFD and CD groups was not statistically different (Figure 2), suggesting that there was no diet-induced obesity in HFD-treated as compared to CD-treated animals.

### 2.2. Triglycerides, Cholesterol, and Glucose Levels in HDF-Treated Animals

Fasted plasma was collected at the end of the study from Tg and WT rats. Cholesterol levels, LDLs, HDLs, triglycerides, and glucose levels were compared. Triglyceride and glucose values were found to be within the expected physiological ranges in the CD and HFD groups (Table S1). Serum cholesterol levels showed similar values between the HFD and CD groups as well as levels of both high- and low-density lipoproteins (Table S1). Our results suggest that the sustained exposure of rats to a PUFA-enriched HFD does not impact metabolic parameters, ruling out a metabolic syndrome.

### 2.3. HFD Did Not Worsen Cognitive Performance in Tg Animals

**Figure 3:**
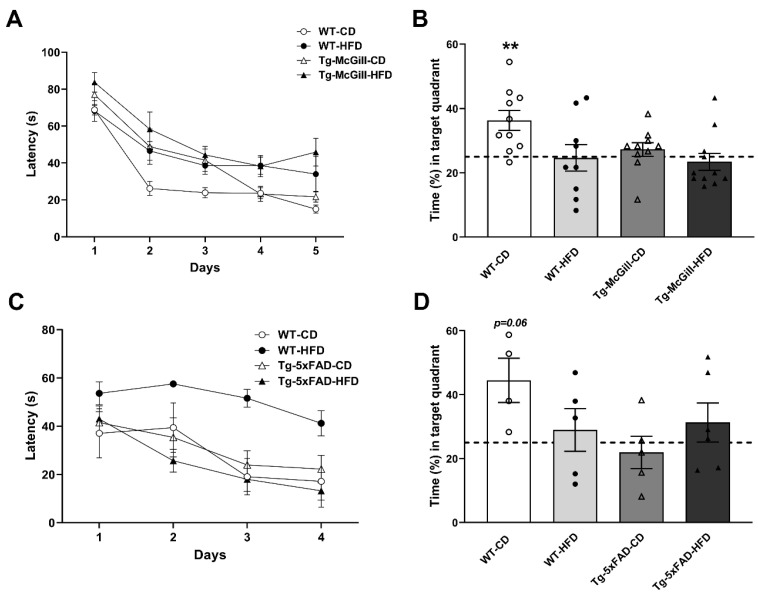
Performance of rats and mice fed with a control diet (CD) or an HFD in the Morris water maze: (**A**) The four groups of rats reduced their escape latencies across the five days of the spatial learning training (factor day: F = 48.3, *p* < 0.001; day × genotype × diet interaction: F = 0.70, *p* = n.s.), indicating that all groups learned the task. (**B**) In the probe trial, the only group that swam significantly more than the 25% of the time (dashed line) that is expected by chance in the target quadrant was the WT-CD group (one-sample *t*-test: *t* = 3.67, ** *p* = 0.005). The remaining three groups swam a similar amount of time in the target quadrant that was not significantly different than expected by chance. This indicates that both the type of diet and the presence of the transgene impaired reference memory, but HFD was unable to worsen learning or reference memory even more in Tg-McGill-HFD rats. WT-CD: 10 rats; WT-HFD: 9 rats; Tg-McGill-CD: 10 rats; Tg-McGill-HFD: 11 rats. (**C**) The four groups of mice exhibited reduced escape latencies across the four days of the spatial learning training (factor day: F = 42.8, *p* < 0.0001). Although it is evident that the WT-HFD group did not learn the task similarly to the other groups, we could not carry out post hoc analyses to confirm this since the interaction day × genotype × diet was not significant (F = 3.04, *p* = n.s.). (**D**) In the probe trial, the WT-CD group showed a statistically strong tendency to swim more than the 25% (dashed line) of the time that is expected by chance (25%, dashed line) (one-sample *t*-test: *t* = 2.81, *p* = 0.06), while the other three groups swam a similar amount of time in the target quadrant that was not different to the amount of time expected by chance. These results suggest that reference memory was impaired in mice, regardless of the genotype or type of diet, although the WT-CD group seemed to remember in which quadrant the platform was; although it did not reach the statistical level of *p* = 0.05, a strong tendency was observed (*p* = 0.06). WT-CD: 4 mice; WT-HFD: 5 mice; Tg-5xFAD-CD: 5 mice; Tg-5xFAD-HFD: 6 mice. Values depicted the mean ± SEM. In (**B**,**D**) individual values are superimposed to the bars.

To shed light on whether an HFD impacts cognition, we exposed all experimental groups to the Morris water maze (MWM). In the case of rats, all groups reduced the escape latencies across the five days of training, indicating that all groups learned the task (Figure 3A). During the probe trial, all groups except for WT-CD showed spatial reference memory impairments (Figure 3B). However, the HFD did not worsen cognitive impairment in Tg-McGill rats (Figure 3B). In mice, all groups reduced the escape latencies across the four days of training (Figure 3C), and similar to the effect observed in Tg rats, the HFD did not worsen cognitive performance in Tg mice (Figure 3D).

### 2.4. There Were No Emergent Phenotypes of Increased Hippocampal Aβ Accumulation in Tg Animals Exposed to HFD

**Figure 4:**
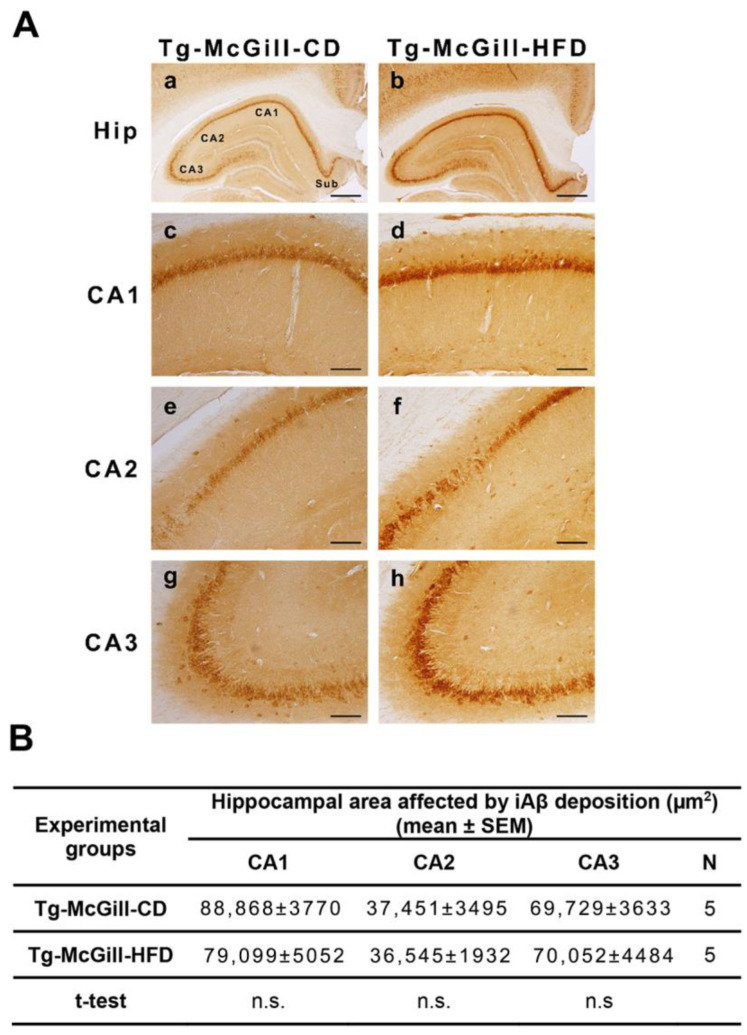
Intraneuronal accumulation of intraneuronal amyloid-beta peptide (iAβ) in Tg-McGill rats fed with a control diet (CD) or a high-fat diet (HFD): (**A**) Representative photomicrographs showing the intraneuronal accumulation of iAβ across the entire hippocampus in (**a**) Tg-McGill-CD and (**b**) Tg-McGill-HFD groups of rats. Scale bars: 600 µm. (**c**–**h**) Representative magnified photomicrographs showing iAβ in selected hippocampal areas (CA1, CA2, and CA3) in (**c**,**e**,**g**) Tg-McGill-CD and (**d**,**f**,**h**) Tg-McGill-HFD groups of rats. Scale bars: 120 µm. (**B**) The area affected by iAβ deposition in CA1, CA2, and CA3 were similar between Tg-McGill rats fed with CD and HFD (CA1: *t* = 1.55, *p* = n.s.; CA2: *t* = 0.23, *p* = n.s.; CA3: *t* = 0.06, *p* = n.s.). Values are expressed as the mean ± SEM of 5 rats per group.

**Figure 5:**
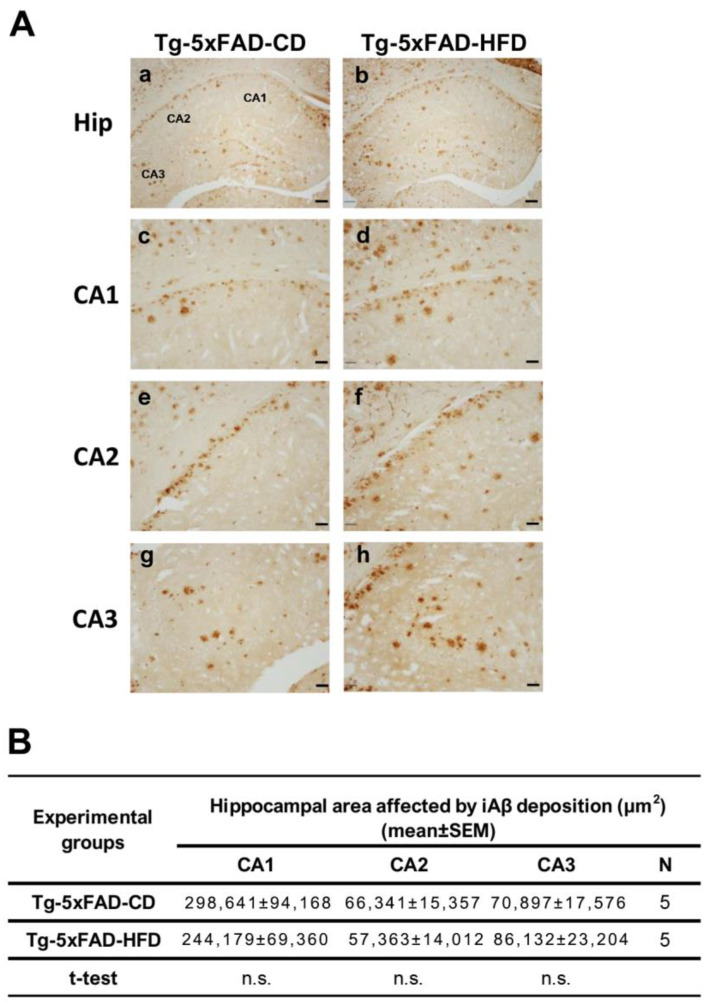
Accumulation of amyloid-beta peptide (Aβ) in Tg-5xFAD mice fed with a control diet (CD) or a high-fat diet (HFD): (**A**) Representative photomicrographs showing the accumulation of Aβ across the entire hippocampus in (**a**) Tg-5xFAD-CD and (**b**) Tg-5xFAD-HFD groups of mice. Scale bar: 100 µm. (**c**–**h**) Representative magnified photomicrographs showing accumulation of Aβ in CA1, CA2, and CA3 in (**c**,**e**,**g**) Tg-5xFAD-CD and (**d**,**f**,**h**) Tg-5xFAD-HFD groups of mice. Scale bars: 40 µm. (**B**) The area affected by Aβ deposition in CA1, CA2, and CA3 were similar between Tg-5xFAD rats fed with CD and HFD (CA1: *t* = 0.44, *p* = n.s.; CA2: *t* = 0.42, *p* = n.s.; CA3: *t* = 0.52, *p* = n.s.). Values are expressed as the mean ± SEM of 5 mice per group.

To determine whether an HFD influences the pathogenesis of AD, we performed a semiquantitative histochemical analysis of hippocampal Aβ immunoreactivity. The affected area by Aβ accumulation did not differ between Tg-CD and Tg-HFD rats (Figure 4A,B). These results were corroborated with a highly sensitive multiplex ELISA, through which the levels of human Aβ isoforms were quantified as 38, 40, and 42 (Figure S1). Moreover, no significant differences were detected in the ratio Aβ42:40 between Tg-CD and Tg-HFD rats (Figure S1), suggesting that APP processing in the brain of Tg rats seems not to be affected by this nutritional paradigm. In addition, no significant variation in the total Aβ accumulation between Tg-CD and Tg-HFD animals was observed in the 5xFAD mouse model using the semiquantitative histochemical analysis of hippocampal Aβ immunoreactivity in all the three analyzed regions (CA1, CA2, and CA3) (Figure 5A,B).

### 2.5. High-Fat Diet Promoted Hippocampal Microglia Activation

**Figure 6:**
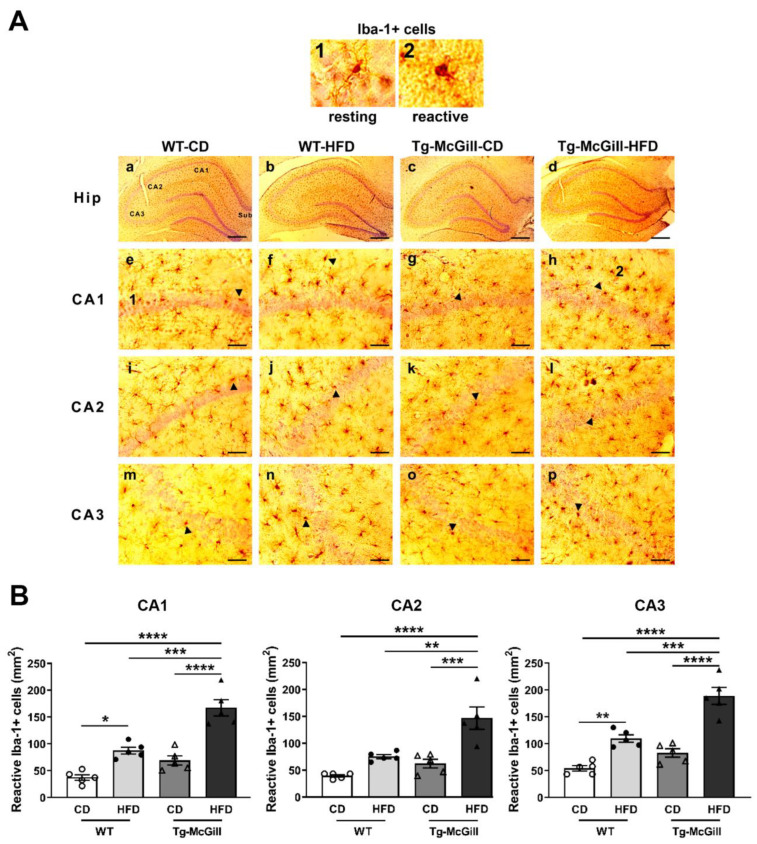
Reactive microglia in WT and Tg-McGill rats fed with a control diet (CD) or a high-fat diet (HDF): (**A**) Upper panel: (**1**) Iba-1-positive cell (taken from (**e**)) in a resting state (small soma and branched morphology) and (**2**) an Iba-1-positive cell (taken from (**h**)) in a reactive state (amoeboid shape, larger soma, and reduced or null processes). Lower panel: (**a**–**d**) Representative photomicrographs of Iba-1-positive cells in the entire hippocampus for the four treatments. Scale bars: 600 µm. (**e**–**p**) Representative magnified photomicrographs of selected hippocampal areas (CA1, CA2, and CA3) for the four treatments. Scale bars: 60 µm. Arrowheads point to Iba-1-positive cells with a morphology indicative of a reactive state. (**B**) Quantification of Iba-1-reactive cells in different areas of the hippocampus (CA1, CA2, and CA3). In the three areas, two-way ANOVA tests revealed that the Tg-McGill-HFD group of rats had a significantly higher number of Iba-1-reactive cells than all other groups (Interaction Genotype × Diet. CA1: F = 6.13, *p* = 0.02; CA2: F = 4.64, *p* = 0.04; CA3: F = 6.75, *p* = 0.01; post hoc Tukey’s multiple-comparison tests: ** *p* < 0.01, *** *p* < 0.001, **** *p* < 0.0001). In addition, in CA1 and CA3 areas, the WT-HFD group of rats had a significantly higher number of Iba-1-reactive cells than the WT-CD group of rats (post hoc Tukey’s multiple-comparison tests: * *p* < 0.05, ** *p* < 0.01); n = 5 rats per group. All values are expressed as the mean ± SEM with individual values superimposed.

**Figure 7:**
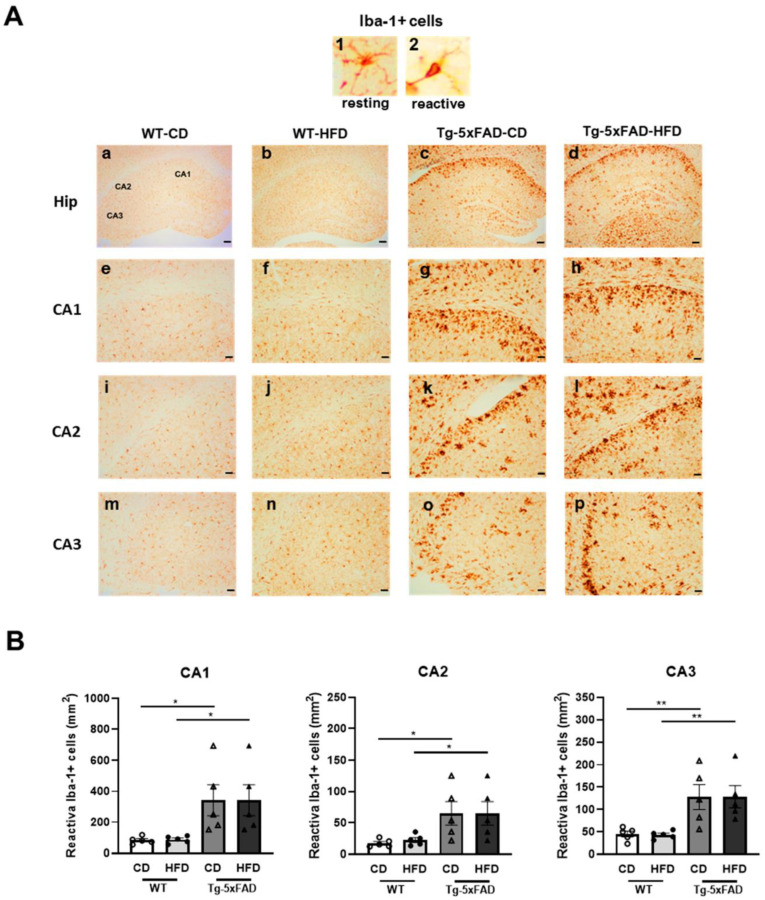
Reactive microglia in WT and Tg-5xFAD mice fed with a control diet (CD) or a high-fat diet (HDF): (**A**) Upper panel: (**1**) Iba-1-positive cell in a resting state (small soma and branched morphology) and (**2**) an Iba-1-positive cell in a reactive state (amoeboid shape, larger soma and reduced or null processes). Lower panel: (**a**–**d**) Representative photomicrographs of Iba-1-positive cells in the entire hippocampus for the four treatments. Scale bars: 100 µm. (**e**–**p**) Representative magnified photomicrographs of selected hippocampal areas (CA1, CA2, and CA3) for the four treatments. Scale bars: 40 µm. (**B**) Quantification of Iba-1-reactive cells in different areas of the hippocampus (CA1, CA2, and CA3). In the three areas, two-way ANOVA tests revealed that the Tg-5xFAD group of mice had a significantly higher number of Iba-1-reactive cells than WT groups (Genotype. CA1: F = 12.936, *p* = 0.002; CA2: F = 11.116, *p* = 0.004; CA3: F = 19.531, *p* = 0.000; post hoc Tukey’s multiple-comparison tests: * *p* < 0.05; ** *p* < 0.01); n = 5 mice per group. All values are expressed as the mean ± SEM with individual values superimposed.

To test whether an HFD raises neuroinflammation, the quantification of Iba-1-positive cells was carried out in the hippocampal sections of rats and mice (n = 5 animals from each experimental group). Iba-1 is a 17 kDa actin-binding protein that is specifically and constitutively expressed in all microglia. It is widely used as an immunohistochemical marker for both branched (inactive) and non-branched (reactive) microglia. Our results in rats showed significant increments in the number of reactive Iba-1-positive cells in most areas of the hippocampus from animals exposed to the HFD as compared to the CD, regardless of the genotype (Figure 6A,B). However, larger increments were observed in Tg rats fed with the HFD (Figure 6A,B). These results may suggest that hippocampal microglia are vulnerable to the detrimental effects of long-term HFD exposure, and the presence of Aβ deposition seems to make microglia even more vulnerable. In addition, correlations between reactive microglia in the entire hippocampus and the area affected by the intraneuronal accumulation of Aβ (CA1, CA2, and CA3) were assessed. No correlation was detected in Tg-CD rats, while in Tg-HFD rats, a significant direct correlation was observed between both parameters (r = 0.887, *p* = 0.044), indicating that HFD-mediated neuroinflammation is associated with cerebral amyloidosis. Differently, the results obtained in the 5xFAD mouse model show a significant increase in Iba-1-reactive cells in CA1, CA2, and CA3 in Tg vs. WT animals, regardless of diet (Figure 7A,B). On the other hand, no significant variation in Iba-1-reactive cells was shown due to HFD exposure in the WT or Tg animals (Figure 7A,B).

As reactive microglia promotes neuroinflammation, in rat hippocampal homogenates, we determined the proinflammatory cytokine levels (IFNγ, TNF-α, and IL-1β); anti-inflammatory cytokine IL-10 in addition to a marker of innate immunity (IL-6); and the chemokine C-X-C motif ligand 1 (CXCL1), which is associated with transendothelial migration of monocytes [26]. The results showed that there were no significant differences for any cytokine among groups except for IFN-γ, in which a significant difference between the WT and Tg genotypes was detected, regardless of the diet (Figure S2).

**Figure 8:**
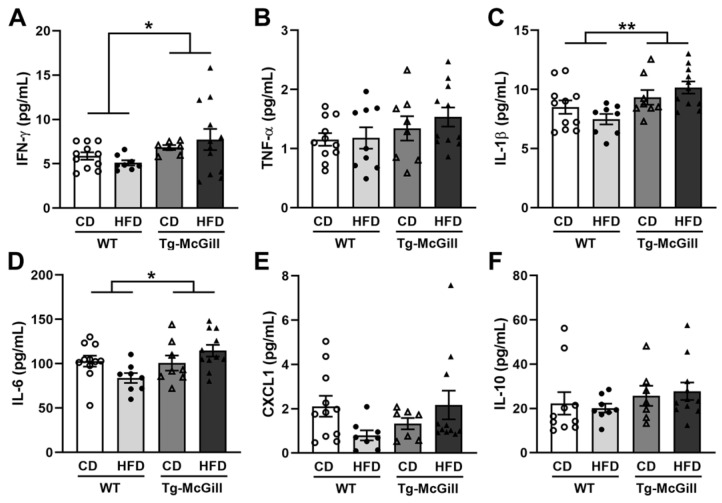
Plasma cytokine and chemokine concentration levels of WT and Tg-McGill rats exposed to a control diet (CD) or a high-fat diet (HFD). For each cytokine and chemokine, a two-way ANOVA test was performed with genotype (WT vs. Tg-McGill) and diet (CD vs. HFD) as between-subject factors. In none of the cases, the interaction genotype × diet was significant, so only the main effects are reported if they were significant. Plasma concentration levels of (**A**) IFN-γ (genotype: F = 4.66, * *p* = 0.04); (**B**) TNF-α; (**C**) IL-1β (genotype: F = 9.97, ** *p* = 0.003); (**D**) IL-6 (genotype: F = 4.40, * *p* = 0.04); (**E**) CXCL1; and (**F**) IL-10. These results indicate that Tg-McGill rats had significantly increased levels of IFN-γ, IL-1β, and IL-6 compared with WT rats, regardless of the type of diet. Data are shown as the mean ± SEM with individual values superimposed. N = 7–12 rats/group. WT, wild-type rats; Tg-McGill, McGill-R-Thy1-APP transgenic rats; CD, control diet; HFD, high-fat diet.

**Figure 9:**
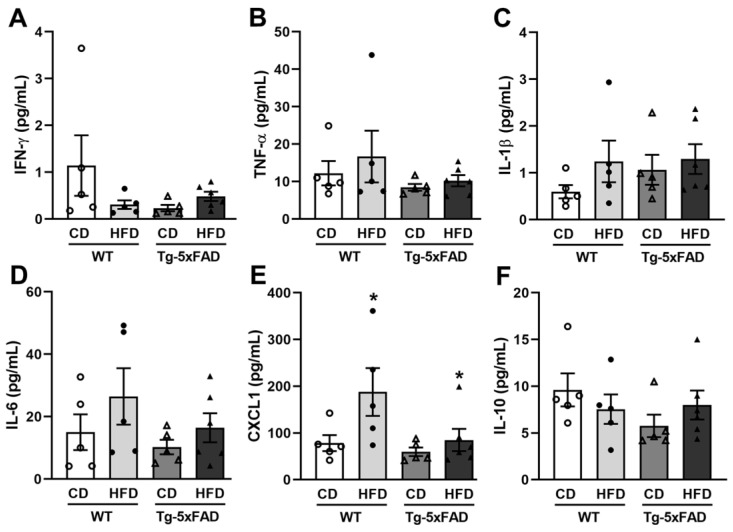
Plasma cytokine and chemokine concentration levels of WT and Tg-5xFAD mice exposed to a control diet (CD) or a high-fat diet (HFD). For each cytokine and chemokine, a two-way ANOVA test was performed with genotype (WT vs. Tg-5xFAD) and diet (CD vs. HFD) as between-subject factors. In none of the cases, the interaction genotype × diet was significant, so only the main effects are reported if they were significant. Plasma concentration levels of (**A**) IFN-γ; (**B**) TNF-α; (**C**) IL-1β; (**D**) IL-6; (**E**) CXCL1 (diet: F = 5.23, * *p* = 0.03); and (**F**) IL-10. These results indicate that HFD induced a significant increase in plasma levels of CXCL1, regardless of the genotype. Data are shown as the mean ± SEM with individual values superimposed. N = 5–6 mice/group. WT, wild-type rats; Tg-5xFAD, 5xFAD transgenic mice; CD, control diet; HFD, high-fat diet.

Since an HFD can alter the immune system [27], which may affect the pathophysiology of AD [28], it is important to measure the plasma levels of proinflammatory cytokines, particularly TNF, as it has been postulated that it can increase the risk of onset and progression of AD [29,30]. In this regard, we determined the plasma levels of IFNγ, TNF-α, IL-1β, IL-6, CXCL1, and IL-10 in all the animal groups analyzed. Our results showed that, in rats, transgenesis promoted the overexpression of IFN-γ, IL-1β, and IL-6, regardless of the type of diet (Figure 8), while in mice, significant increments were detected in CXCL1 levels only in animals exposed to the HFD, regardless of the genotype (Figure 9).

### 2.6. Diet-Related Expression of Nitro-Oxidative Stress Parameters in Mice and Rat Brains

**Figure 10:**
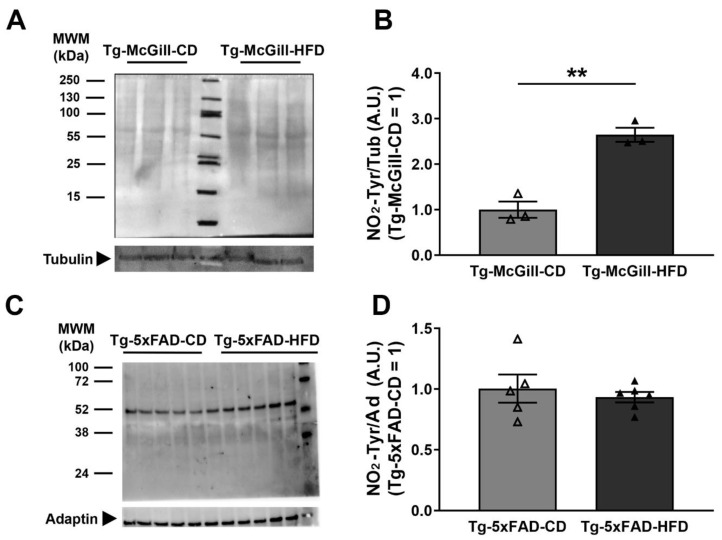
Levels of protein tyrosine nitration (NO_2_-Tyr) in Tg-McGill rats and Tg-5xFAD mice fed with a control diet (CD) or a high-fat diet (HFD). Representative Western blot of hippocampal homogenates of (**A**) Tg-McGill-CD (n = 3) and Tg-McGill-HFD (n = 3) rats probed with anti-NO_2_-Tyr and anti-tubulin (molecular weight of tubulin 50 kDa). (**B**) The Tg-McGill-HFD group of rats had significantly higher levels of protein tyrosine nitration levels than the Tg-McGill-CD group of rats (*t* = 6.94, ** *p* = 0.002). (**C**) Representative Western blot of hippocampal homogenates of Tg-5xFAD-CD (n = 5) and Tg-5xFAD-HFD (n = 6) mice probed with anti-NO_2_-Tyr and anti-adaptin (molecular weight of adaptin 104 kDa). (**D**) No differences were observed between the mice groups (*t* = 0.62, *p* = n.s.). In rats and mice, the optical density (O.D.) of all bands from approximately 100 kDa to 25 kDa was quantified and normalized to the O.D. of tubulin or adaptin. In the Western blot of mice (**C**), the quantification of only the most intense band (approximate molecular weight 60 kDa) also showed no difference between the groups (*t* = 2.13, *p* = n.s.). Each lane in each membrane represents an individual animal, and technical replicates (n = 3) were conducted, with the mean value for each animal represented by the points in panels (**B**,**D**). All values are expressed as the mean ± SEM with individual values superimposed.

To assess if the type of diet unbalances nitro-oxidative stress, using Western blot, we measured the levels of protein nitration (NO_2_-Tyr) in the hippocampal homogenates. As depicted in Figure 10, a significant increase was observed in the hippocampal homogenates of HFD-fed Tg rats but not in HFD-fed Tg mice. Furthermore, in addition to NO_2_-Tyr levels, we determined the concentration of ADMA (**^•^**NO inhibitor) and the activity of GS in Tg rat brains via HPLC-MS/MS. The results showed no significant differences between experimental groups exposed to the CD or HFD; however, a trend of an increase in NO_2_-Tyr was observed in the brain homogenates of Tg-HFD as compared to Tg-CD rats (Table S2). Indeed, protein NO_2_-Tyr values in the brain homogenate samples from the rat model ranged from 26.6 ± 3.3 to 39.2 ± 5.4 µmol/mol Tyr. There are very few reports with the quantitation of NO_2_-Tyr levels in tissues. The reported values are in the order of NO_2_-Tyr detected in lung tissue (79 ± 8 µmol/mol Tyr) [31].

## 3. Discussion

HFD rodent models have contributed significantly to the analysis of the pathophysiology of lifestyle diseases; however, experimental evidence shows that in order to carry out useful clinical strategies for the human population, it is necessary to perform more detailed studies in preclinical models to reveal the precise role that dietary fats play in the pathophysiology of AD [17]. In our previous study performed in young rats exposed for a long period to a high-energy diet (39% kcal from fat based on lard) and supplemented with fructose (10% *w*/*v* in drinking water) [32], we showed the negative impact of the Western diet on neuropathology and cognition. Therefore, here, we sought to test if dietary fat intake (40% kcal from fat from corn oil) without fructose supplementation was also able to modulate cognition and AD-related neuropathology. Moreover, to evaluate the hypothesis that fat intake may have a different impact depending on the age and the stage of the disease, in this report, we started the experiment with young Tg-McGill rats or adult Tg-5xFAD (C57BL/6J) mice, both preclinical models of early and late AD-like neuropathology, respectively.

Taking into account the wide range of commercially available purified diets, we decided not to use an in-house-formulated diet in this study in order to guarantee homogeneity and minimal loss of labile nutrients as recommended [33]. Usually, the dietary ingredients in commercial diets provided by reputable suppliers are of the highest quality, and several analyses of each ingredient are available. Here, we compared the performance of two rodent diets formulated by the American Institute of Nutrition (AIN): AIN-93G (8.4:1 omega-6-to-omega-3 ratio) and AIN-76A (25:1 omega-6-to-omega-3 ratio). AIN-93G is the growth diet currently recommended by the AIN as a revision of AIN-76A, which has been broadly employed worldwide but displays nutritional concerns because of its higher fat content. The major differences between AIN-93G with AIN-76A are as follows: The 7 g soybean oil/100 g diet was replaced with a 5 g corn oil/100 g diet in order to increase the amount of linolenic acid, essential to make hormones that regulate the immune and central nervous systems; the fat amount was reduced from 20% in AIN-76A to 7.1% in AIN-93G; the sucrose levels were reduced (because of adverse effects) and carbohydrates were replaced with starch; L-Cys substituted DL-methionine (to supplement casein, which is deficient in sulfur amino acids); the amount of vitamins E, K, and B-12 were augmented, and vanadium, lithium, boron, nickel, fluoride, silicon, and molybdenum were included to the mineral mix.

At the end of the nutritional paradigm, the overall weight gain was similar between the animals exposed to the HFD (40% kcal from fat) vs. CD (16.4% kcal from fat), suggesting that the diet was not obesogenic. Moreover, the values of fasting triglycerides and glycemia from rat blood were compatible with the range of normal values according to the fasting blood parameters for male albino rats (glycemia: 90–130 mg/dL and triglycerides: 123–186 mg/dL) [34], indicating that although there were significant increases in Tg rats exposed to the HFD, these values were not compatible with a metabolic syndrome.

It was reported that a rodent HFD containing ≈45% kcal from fat generates obesity and metabolic syndrome compared with CD (3–18% kcal from fat) [35,36]. However, in this report, the animals exposed to the HFD did not develop obesity or a metabolic syndrome. We ruled out the possibility that the negative result was a consequence of a short exposure time, taking into account that general protocols to place mice on an HFD treatment suggest starting when mice are 10 weeks of age, and generally treatment lasts 12 weeks (http://bridgeslab.sph.umich.edu/protocols/index.php/High_Fat_Diet_Treatment. Accessed on 25 October 2023). In the case of rats, the protocols for chronic exposure start with 6–8 week-old animals, and treatment lasts 2–3 months. In this report, we extended the nutritional paradigm up to 6 months in rats to meet the age at which McGill-R-Thy1-APP animals start displaying amyloid plaque deposition (6–9 months old) to assess the impact of diet on neuropathology.

Classical HFD effects on obesity and metabolic syndrome were observed only in diets based on lard and olive oil [37]. Here, the fat source was corn oil. The major lipid constituents in lard-based HFDs are saturated fatty acids (SFAs), while in corn oil-based HFDs, the major lipid constituents are polyunsaturated fatty acids (PUFAs). In agreement with our results, there is convincing evidence that when polyunsaturated fats replace saturated fats in the diet, this could help to reduce serum very low-density and low-density lipoprotein concentrations because the liver preferentially converts PUFAs into ketone bodies instead of triglycerides [38]. Regarding PUFA intake, an omega-6-to-omega-3 ratio between 2:1 and 1:1 is the recommended ratio for human nutrition. In Western diets, the omega-6-to-omega-3 ratio has increased to between 10:1 and 20:1. In our experimental paradigm, the HFD group had an elevated omega-6-to-omega-3 ratio as compared to the CD group (25:1 vs. 8.4:1).

Notably, most of the reports showing obesity associated with increased brain oxidative stress and neuroinflammation were performed by exposing rodents to a high-fat/high-sugar diet, highlighting fructose as a relevant player in brain dysfunction. Gut- and liver-derived metabolic intermediates of fructose or fructose itself can reach the brain, impairing insulin signaling and promoting brain inflammation, mitochondrial dysfunction/oxidative stress, cognitive deterioration, and the onset of mechanisms related to neurodegeneration [39].

Strikingly, in our experiments, after sustained exposure to the HFD, adult animals from background strains showed slight impairments in cognitive performance assessed with the MWM test, while transgenic rodents did not exhibit a worsening effect in their characteristic memory deterioration. The effect on cognition in rodents exposed for a long period to an HFD remains debatable, and differences may be considered by diet composition, time of exposure, age and gender of the animals, and/or genetic differences between individual specimens [40,41,42,43,44,45,46]. According to the reported findings in rodent strains, the strong effects of HFD treatment on cognitive performance were observed in both young and adolescent animals; however, a small number of studies employed adult animals [47].

Here, we showed that adult mice (3 months old) exposed to an HFD for 2.5 months exhibited a decrease in their performance in learning and memory. The cognitive deterioration observed in the background strains and the increased expression of Iba-1-positive cells in the hippocampi of WT rats exposed to the HFD support the hypothesis that HFDs induce negative behavioral effects in part by modulating the activity of microglia. In this regard, an HFD seems to trigger a “basal neuroinflammatory tone” that ends up being responsible for cognitive deterioration. Microglia are activated following exposure to pathogen-associated molecular patterns and/or endogenous damage-associated molecular patterns. Studies have demonstrated the ability of proinflammatory cytokines (IL-6 and TNF-α) to induce activation, increasing microglial cell proliferation and the production of microglia-derived proinflammatory molecules. In addition, it has been proposed that peripheral proinflammatory molecules are able to enter the brain through regions lacking the blood–brain barrier (BBB) or through various mechanisms, including the fenestrated capillaries of the BBB, cytokine-specific transporters, or by increasing the permeability of the BBB [48]. Moreover, peripheral cytokines have the ability to activate the sensory fibers of peripheral nerves, leading to increased cytokine levels in the brain [49]. The difference observed in Iba-1 activation between the two Tg models exposed to the HFD may depend on the impact that HFD exposure has on peripheral proinflammatory cytokine levels and most importantly on the fact that Tg rats do not display amyloid plaques. 

In contrast to previous reports showing that HFDs impact APP processing, aggravating neuropathology by enhancing the β-site cleavage of APP and potentiating Aβ accumulation in the brain [50], in this study, we did not observe increases in the levels of any of the three human Aβ isoforms (38, 40, and 42) in the hippocampus, suggesting that the intake of fat from corn oil does not modulate APP metabolism as previously reported in diets that revealed an increase in cholesterol levels [51].

Increasing evidence suggests that Aβ-mediated microglia activation is involved in neurotoxicity through the release of proinflammatory cytokines (IFNγ, TNF-α, and IL-1β) [52]. In this work, similar to what was shown in previous reports [53], we were able to observe significant differences in the levels of IFN-γ in the brain homogenates of Tg vs. WT animals. IFN-γ is a potent molecule normally expressed by glia and neurons, and it exhibits immunoregulatory functions within the central nervous system compartment, including the activation of microglia and the stimulation of macrophages to release toxic oxygen radicals [54]. By contrast, no significant differences were detected in the hippocampal levels of TNF-α or IL-1β. Notably, we did not detect significant differences in the brain homogenates of animals exposed to the HFD as compared to the CD. Our interpretation is that HFD-induced microglial activation is not strong enough to generate the required increments of proinflammatory cytokines able to be detected in brain homogenates. 

Regarding the impact of HFDs on peripheral inflammation, our results support the notion that transgenesis in rats promotes the overexpression in the plasma of IFN-γ, IL-1β, and IL-6 regardless of the type of diet, as well as the concept that in Tg rats, the blood–brain barrier (BBB) became more permeable probably due to cerebral amyloid deposition but an HFD based on corn oil did not lead to an increase in peripheral inflammation that could be detected with a high sensitive ELISA test. Therefore, increased plasma levels of IL-6 may be one of the mechanisms that contribute to the presence of an increased population of reactive microglia in the hippocampus of Tg rats.

By contrast, in mice, significant increases were detected in the plasma levels of CXCL1 only in animals exposed to the HFD, regardless of the genotype. This experimental evidence suggests that in mice, an HFD based on corn oil seems to be detrimental, taking into account the fact that CXCL1 induces the phosphorylation and caspase-3-dependent truncation of tau protein, which is an early neuropathological event in AD [55]. It is worth noting that 5xFAD mice develop NFT and it could be possible that this kind of HFD may promote AD-related neuropathology in longer exposures and at more advanced ages.

The lack of an evident impact of this kind of HFD on the brain levels of biomarkers of nitro-oxidative stress suggests that the three parameters analyzed in this report (the protein levels of NO_2_-Tyr and ADMA and GS activity) can be modulated by diets based on lard and olive oil supplemented with fructose and that an energy increase from fat based on corn oil has no effect. Still, the HPLC MS/MS analysis provided herein for protein NO_2_-tyrosine and ADMA levels and the enzymatic assay for GS in the brain samples provide a foundation for future quantitative studies on the impact of nitro-oxidative stress in Alzheimer’s disease and other neurodegenerative diseases [12]. 

This is a descriptive report with several limitations. First, our study in rats was limited to only males, so these experiments would need to be repeated in females as well before our findings could be generalized to both sexes. Second, our study only examined one type of hippocampal-dependent memory task. The Morris water maze was chosen for this study because a previous report from our own laboratory showed that it is sensitive to HFDs [32]. Finally, we did not examine potential mechanisms underlying HFD-induced hippocampal dysfunction.

## 4. Materials and Methods

### 4.1. Animals and Ethical Implications

McGill transgenic (Tg) rats were provided to the Leloir Institute Foundation (FIL) by The Royal Institution for the Advancement of Learning/McGill University, Canada, through the signing of an MTA FIL (Argentina) and McGill University (Canada). Tg rats express human APP751 with the Swedish and Indiana mutations, under the control of the murine Thy1.2 promoter [20]. All experimental procedures were carried out in agreement with the guidelines of ARRIVE and OLAW-NIH. The protocol was approved by the local animal care committee (CICUAL # A5168-01). Briefly, 5xFAD transgenic mice overexpressing human APP with three FAD mutations (Swedish (K670N, M671L), Florida (I716V), and London (V7171)) and human PSEN1 with two FAD mutations (M146L and L286V) [21] were maintained at the IBIMA by crossing heterozygous transgenic mice with B6/SJL F1 breeding mice from The Jackson Laboratory in Bar Harbor, ME, USA. Prior to the study, the Research and Clinical Ethics Committee of the Regional University Hospital of Malaga and the University of Malaga approved the protocol. Each of the experimental procedures on animals strictly adhered to the ARRIVE guidelines and conformed to the EU Directive 2010/63/EU for animal studies.

Background strains were Wistar for Tg rats and B6/SJL for Tg mice. The animal facilities at the FIL and IBIMA were controlled for temperature (22 C ± 2), relative humidity 40%, and lighting (12 h light/dark cycle). Water and diets were provided ad libitum.

### 4.2. Diets

Purified diets were provided by TestDiet (Tucson, AZ, USA). The control diet (CD) (AIN-93G) contains 18.3% protein, 7.1% fat, 5% fiber, and 63.2% carbohydrates (energy from fat based on soybean oil: 16.4%), and it is recommended by the American Institute of Nutrition (Rockville, MD, USA) for rodent growth [33]. The high-fat diet (HFD) (AIN-76A) contains 17.4% protein, 20% fat, 5% fiber, and 49.9% carbohydrates (energy from fat based on corn oil: 40.1%). Both diets are balanced in protein, vitamins, and essential minerals. The omega-3-to-omega-6 ratio is 8.4:1 for AIN-93G and 25:1 for AIN-76A. All diets were fed as pellets.

### 4.3. Nutritional Protocol

Rats (15 Tg and 15 WT) and mice (5 Tg and 5 WT) were housed individually and allowed to consume the CD and water ad libitum, while the other 2 groups of animals (15 Tg rats and 15 WT rats; 6 Tg mice and 5 WT mice) consumed the HFD. We exposed young rats (2 months old) and adult mice (3 months old) to the nutritional paradigm for 6 and 2.5 months, respectively. At the end of the experiment, rats were approximately 8 months old, while mice were 5.5 months old.

### 4.4. Ante-Mortem Observations

All animals were observed twice daily for mortality, gross motor activity, behavioral changes, and alterations in appearance. Food consumption and individual body weights were recorded on a weekly basis, until the conclusion of the experiment. We calculated weight gain by subtracting the final weight at the conclusion of the nutritional exposure from the initial weight and presented it as the total weight gain (in grams).

### 4.5. Behavioral Assessment

Behavioral tests for rats and mice were conducted in the behavioral facilities at the FIL and IBIMA, respectively. The chosen behavioral test was the Morris water maze (MWM). With this test, we evaluated spatial learning and spatial reference memory. The apparatuses consisted of two circular water pools: one for rats (180 cm in diameter) and one for mice (150 cm in diameter). Both water pools were filled with water (rats: 22 ± 1 °C; mice: 24 ± 1 °C), and each one included a circular goal platform to allow animals to escape from water (rats: 10 cm in diameter; mice: 11 cm in diameter). In the case of rats, the water pool was painted black, and the circular goal platform was made of transparent acrylic plastic, making it invisible when it was submerged 2 cm from the water’s surface, while in the case of mice, the circular goal platform was made invisible by making water opaque with non-toxic white paint. The two water pools were divided into four equal imaginary quadrants ((north (N), east (E), south (S), and west (W)), and four possible starting positions (located in the peripheral region of each of the four imaginary quadrants) were employed to release the rats/mice into the water. Finally, both water pools were located in experimental rooms with visual cues surrounding the pools and hanging on the walls. These cues served as distal extra-maze cues to facilitate spatial orientation. We followed the same protocols carried out previously [32,56,57,58], with few modifications. The entire protocol included three/four phases: (a) habituation phase; (b) visible platform training; (c) spatial learning training; and (d) probe trial. Habituation phase: The habituation phase was performed in mice but not in rats since, in our experience, rats adapt to the protocol very quickly, and the addition of a habituation phase does not make any difference in their performance. The habituation phase in mice consisted of releasing mice in the water pool in the absence of the goal platform and letting them swim for 60 s. Each mouse received two habituation trials (inter-trial interval: 15 min) during a single day. Visible platform training: Both rats and mice were submitted to the visible platform training. For both species, the goal platform was made visible by reducing the water level and allowing the goal platform to protrude 2 cm from the water surface. In addition, a vertically standing 50 mL black polystyrene tube was attached to each goal platform to enhance visibility. Rats and mice were submitted to four daily sessions (inter-trial interval for rats: 20–30 min and for mice: 15 min) for two days. The platform was situated in the center of the escape quadrant during each session, and the platform’s position was changed to four quadrants during the same day. The starting positions were also alternated. The rats and mice were released into the water pool, and the latency to the platform was recorded. If animals did not find the platform once 120 s (rats) or 60 s (mice) elapsed, they were guided to the platform by the experimenter. In these cases, the 120 s (rats) and 60 s (mice) periods were recorded as the latency to reach the platform. Both rats and mice successfully reduced their escape latency across trials and days, suggesting that wild-type and transgenic groups, regardless of the diet, were visually unimpaired, and their swimming ability was preserved (data not shown). Spatial learning training: This procedure was similar to the visible platform training, but the goal platform was made invisible, as mentioned before. Rats and mice were submitted to four daily sessions (inter-trial interval for rats: 20–30 min, and for mice: 15 min) for five (rats) or four (mice) days. The latency to reach the goal platform was recorded, and if the animal failed to reach the platform after 120 s (rats) or 60 s (mice), it was guided to the platform by the experimenter, and the maximum latency (120 s for rats and 60 s for mice) was recorded. Probe trial: Twenty-four hours after the last spatial learning trial, the probe trial was performed. The platform was removed from the water pool, and the animals were released from one starting point not used before and left to search for the absent platform for 60 s. The time spent in the four quadrants was recorded.

In the case of rats, 11 animals were randomly selected to perform the water maze task, but some of the rats learned to escape from the pool by jumping from the goal platform during the visible platform training. These rats were excluded from subsequent phases. Therefore, the final number of rats that performed the entire water maze protocol was 10 WT-CD, 9 WT-HFD, 10 Tg-McGill-CD, and 11 Tg-McGill-HFD. In the case of mice, one mouse from the WT-CD group learned to escape from the pool by jumping from the goal platform during the visible platform training and therefore was excluded from further training. Thus, the final number of mice that performed the entire water maze protocol was 4 WT-CD, 5 WT-HFD, 5 Tg-5xFAD-CD, and 6 Tg-5xFAD-HFD.

### 4.6. Blood and Brain Isolation

Rats were anesthetized via the inhalation of isoflurane and euthanized via decapitation with a guillotine. Ten milliliters of blood were collected in sterile tubes containing ACD buffer (39 mM citric acid, 75 mM sodium citrate, 135 mM dextrose, pH 7.4). The blood was centrifuged, and the plasma was stored at −80 °C in aliquots. The brain was isolated following standard procedures, and the right hippocampus was rapidly separated and stored at −80 °C for biochemical studies. The left half of the brain was rapidly removed, immersed first in 4% paraformaldehyde for two days at 4 °C and then in 30% sucrose solution, and finally serially sectioned at 30 µm for immunohistochemistry. Similar procedures were followed in mice with the following minor modifications: (1) mice were euthanized with sodium pentobarbital (150 mg/kg, i.p.), and (2) the left hemisphere was cut in coronal sections of 25 μm for immunohistochemistry.

### 4.7. Brain Homogenates and Western Blotting (WB)

Brain tissue homogenates for ELISA and Western blotting (WB) were prepared in RIPA buffer in the presence of protease inhibitors and phosphatase inhibitors. Protein concentration was determined using a Pierce MicroBCA Protein Assay Kit (Thermo Scientific, Boston, MA, USA). For WB, the samples were run in SDS–Tricine gels and transferred to PVDF membranes. The membranes were blocked with 5% skim milk or BSA in PBS-T (0.1% Tween-20 in PBS) at R.T. for 1 h. Then, they were incubated (overnight) with rabbit anti-3-nitrotyrosine (1:1000, Upstate) (rats) or with mouse monoclonal anti-3-nitrotyrosine (1:500, Life Technologies, 32-1900, Carlsbad, CA, USA) (mice). Peroxidase-conjugated secondary antibodies and an enhanced chemiluminescence detection system (ECL, Thermo Scientific, Waltham, MA, USA) were employed to detect immunoreactivity and quantify with ImageQuant™ LAS4000 (GE Healthcare, Chicago, IL, USA).

### 4.8. Immunohistochemistry

Brain sections from 5 randomly chosen rats/group were analyzed as previously described [20]. Aβ deposition was detected with McSA1 mouse monoclonal antibody (1:1000; Montreal, QC, Canada) [59] and visualized with 0.05% diaminobenzidine/0.01% hydrogen peroxide (Vector Laboratories, Burlingame, CA, USA) using an Olympus BX50 microscope (Tokyo, Japan) [57]. In each of the five randomly selected rats, we chose three coronal sections distributed equidistantly along the dorsal hippocampus (−2.30 to −3.8 mm from bregma according to the Paxinos and Watson’s rat brain atlas [60]). In each coronal section, we took one microphotograph approximately in the middle of the CA1 area, one in the CA2 area, and one in approximately the middle of the CA3 area. Then, in all the microphotographs taken, we delineated the area affected by Aβ deposition and measured the size of the affected area with ImageJ (NIH, Bethesda, MD, USA) (expressed as square micrometers). Next, in each animal, we averaged the area affected by Aβ deposition in CA1, CA2, and CA3. Finally, we calculated the group mean by averaging the values of the 5 animals. The expression of microglia was observed with Iba-1 rabbit polyclonal antibody (1:500; Wako Chemicals USA, Inc., Richmond, VA, USA) and visualized with 0.05% diaminobenzidine/0.01% hydrogen peroxide (Vector Laboratories, Burlingame, CA, USA). Counterstaining with cresyl violet was performed. Microphotographs of CA1, CA2, and CA3 areas from the dorsal hippocampus were taken using an Olympus BX50 microscope (Tokyo, Japan), and four slides per rat were analyzed (n = 5 rats/group). The total number of reactive Iba-1-positive cells (amoeboid shape, larger soma, and reduced or null processes) was counted in each microphotograph using ImageJ (NIH, Bethesda, MD, USA) and expressed as reactive Iba-1-positive cells per mm^2^ [25]. On the other hand, brain sections from 5 randomly chosen mice/group were analyzed by using systematic random sampling. Every sixth brain section (240 μm apart) was stained using a free-floating immunohistochemistry procedure. Aβ deposition was detected with an anti-β amyloid antibody (MOAB-2) monoclonal antibody (1:500; Abcam; ab126649; Cambridge, UK). Sections were washed at R.T., blocked, and later incubated with primary antibody overnight. Then, the sections were incubated with the secondary antibody (biotinylated goat anti-mouse (1:500, GE Healthcare, Chicago, IL, USA) and revealed using the peroxidase-conjugated ExtraAvidin method and diaminobenzidine. Immunohistochemical images of amyloid-β were acquired with digital Camera DP70 (Olympus Iberia, S.A., Barcelona, Spain) connected to a microscope Olympus BX41 (Tokyo, Japan). The area affected by Aβ deposition was measured and calculated similarly to the procedure for rats, as described above, with 3 coronal sections for mice. The coordinates of the dorsal hippocampus (−1.46 to −2–18 mm from bregma) were taken from Franklin and Paxinos’s mouse brain atlas [61]. The expression of microglia was detected with Iba-1 rabbit polyclonal antibody (1:1000; Wako Chemicals USA, Inc., Richmond, VA, USA). Sections were washed at R.T., blocked, and later incubated with primary antibody overnight. Then, the sections were incubated with the secondary antibody (biotinylated goat anti-rabbit (1:500, GE Healthcare, Chicago, IL, USA) and visualized with the same method used for amyloid-β. Immunohistochemical images of Iba-1 were acquired with a DP70 digital camera (Olympus Iberia, S.A., Barcelona, Spain) connected to an Olympus BX41 microscope (Tokyo, Japan). Microphotographs of hippocampal CA1, CA2, and CA3 areas were taken, and three slides per mouse were analyzed. The total number of reactive Iba-1-positive cells (amoeboid shape, larger soma, and reduced or null processes) was counted in each microphotograph using ImageJ (NIH, Bethesda, MD, USA) and expressed as reactive Iba-1-positive cells per mm^2^ [25].

### 4.9. Cytokine Panel Assay

To quantify proinflammatory cytokines, MSD^®^ (MesoScale Discovery, Gaithersburg, MD, USA); V-PLEX Proinflammatory Panel 1 (mouse) Kit (10 spots: IFN-γ, IL-1β, IL-2, IL-4, IL-5, IL-6, IL-10, KC/GRO (also known asCXCL1), IL-12p70, and TNFα); and V-PLEX Proinflammatory Panel 2 (rat) Kit (9 spots: IFN-γ, IL-1β, IL-4, IL-5, IL-6, IL-10, IL-13, KC/GRO (also known as CXCL1), and TNFα) were used following the manufacturer’s instructions. Briefly, plasma (diluted 1:2) or hippocampal homogenates (approx. 0.5–1.25 ug/well) in 25 μL were loaded onto MULTI-SPOT^®^ microplates (in duplicates) and detected with SULFO-TAG™-labeled detection antibody. The light that was emitted via electrochemical stimulation was read with an MSD QuickPlex SQ120 instrument. MSD Workbench 4.0 software was employed to analyze the data. 

### 4.10. Human Aβ Species Assay

A V-PLEX PLUS Aβ Peptide Panel 1 Kit was employed to determine soluble human Aβ 38/40/42 levels, as instructed by the manufacturer (MesoScale Discovery, Gaithersburg, MD, USA. Briefly, hippocampal homogenates were loaded in duplicate onto MULTI-SPOT^®^ microplates, which had been precoated with antibodies specific to the C-termini of Aβ38, Aβ40, and Aβ42. Detection was performed using SULFO-TAG™-labeled 6E10 antibody. The light emitted upon electrochemical stimulation was then read using the MSD QuickPlex SQ120 instrument, and the data were analyzed using MSD Workbench 4.0 software.

### 4.11. Sample Preparation for 3-NO_2_-Tyrosine and ADMA Quantification in the Hippocampus

Following euthanasia, the hippocampus samples were frozen and lyophilized until analysis. For sample preparation, dried samples were weighed, hydrated in PBS buffer, homogenized with ZrO beads, and centrifuged at 14,000× *g* for 30 min at 4 °C. Then, the homogenates were transferred to another tube, ADMA-d6 (Cayman Chemicals, Ann Arbor, MI, USA) was added as an internal standard, and cold acetone was incorporated for protein precipitation. The mixture was centrifuged at 14,000× *g* for 30 min and the pellet and supernatant were separated using 3-NO_2_-tyrosine and ADMA analysis, respectively. In the case of the supernatant, it was transferred to another tube, evaporated until dry, and stored at −20 °C. On the day of analysis, these samples were resuspended in deionized water plus 0.1% formic acid for HPLC-MS/MS analysis. In the case of the pellet, sample processing was carried out as previously described [62]. Briefly, the pellet was washed with cold acetone and centrifuged at 4500 g for 10 min, and this procedure was repeated twice. Then, acetone was evaporated with nitrogen gas, [^13^C_9,_^15^N_1_]-Tyr [^13^C_6_]-NO_2_Tyr (Cambridge Isotope Laboratories, Inc., Tewksbury, MA, USA) were added as internal standards, the pellets were resuspended in methanesulfonic acid with tryptamine, and hydrolysis vials were exposed to cycles of vacuum and argon infusion to reduce oxygen exposure during incubation at 110 °C overnight. The next day, the pH of the samples was neutralized using NaOH and enriched using Strata-X 33 µ Polymeric Reversed Phase columns prior to NO_2_Tyr/Tyr quantification via HPLC-MS/MS.

### 4.12. Quantification of Nitro-Oxidative Stress

Protein nitration (NO_2_-Tyr) levels in the brain homogenate samples were assessed by HPLC-MS/MS, using a reverse phase column and a mass spectrometer (QTRAP4500, ABSciex, Framingham, MA, USA) in MRM (multiple reaction monitoring) mode. A reversed-phase column (Prodigy ODS(2), 5 μm, 150 × 2.0 mm, Phenomenex, Torrance, CA, USA) was employed to separate samples by gradient elution with formic acid 0.1% in nanopure H2O (*w*/*w*) (Solvent A) and in acetonitrile (*w*/*w*) (Solvent B). The elution gradient was from 5% B to 40% B over 12 min at 200 µL/min, 30 °C, and prior to each injection, wash and equilibration of column was performed. We set the ion source and gas parameters as follows: IS:5500 V; TEM: 500 °C; GS1: 30; GS2: 30; CUR: 50. The analyzer was set in positive MRM mode, that included 2 transitions/analyte: 3-NO_2_[^12^C_6_]Tyr (*m*/*z* 227/181, and 210); 3-NO_2_[^13^C_6_]Tyr (*m*/*z* 233/187, and 216); [^12^C_6_]Tyr (*m*/*z* 182/136, and 165); and [^13^C_9_,^15^N_1_]Tyr (*m*/*z* 192/145, and 174). To minimize tyrosine oxidation during the processing of the sample, we performed acid hydrolysis in presence of tryptamine and under vacuum in order to prevent O_2_-dependent processes. However, we analyzed artefactual nitration by also monitoring the nitrated tyrosine internal standard [3-NO_2_[^13^C_9_,^15^N_1_]Tyr (*m*/*z* 237/190, and 219)] [63]. We calculated the content of tyrosine and 3-nitrotyrosine in samples according to the area ratio to the corresponding internal standard and corrected after adjusting for the level of artefactual generation (it was controlled to be <10%).

ADMA levels were quantified via HPLC-MS/MS, using isocratic elution in two normal-phase columns arranged in series (Beckman Ultrasphere Silica (5 μm, 2.0 × 250 mm) and Vydac 101TP54 Unbonded Silica (5 μm 4.6 × 250 mm) and the mass spectrometer (QTRAP4500, ABSciex, Framingham, MA, USA) in MRM mode. Elution was performed with ammonium formate 10 mM plus 0.1% v-v formic acid in nanopure H_2_O at 300 µL/min flow rate. Ion source and gas parameters were set as follows: IS:4500 V; TEM: 500 C; GS1: 32; GS2: 20; and CUR: 27. The analyzer was set in positive MRM mode including three transitions per analyte; ADMA (*m*/*z* 203/70, 46 and 172), and D_6_-ADMA (*m*/*z* 209/70, 52 and 164). ADMA concentration in samples was calculated employing a calibration curve containing both natural and isotopically labeled standards.

### 4.13. Glutamine Synthetase (GS) Activity

The GS activity was measured with the ɣ-glutamyltransferase assay, which is based on the synthesis of ɣ-glutamylhydroxamate from glutamine and hydroxylamine, in the presence of manganese, arsenate, and ADP: L-glutamine + NH_2_OH → ɣ-glutamylhydroxamate + NH_3_, as described in [12]. Once the ɣ-glutamylhydroxamate was formed, it was treated with FeCl_3_, and the formation of the complex [Fe_3_^+^-ɣ-glutamylhydroxamate] was measured via absorbance at 500 nm. To preserve the endogenous state of lipid oxidation, butyl hydroxytoluene was added to a 0.01% (*v*/*v*) solution immediately after separation from the blood to prevent the ex vivo generation of isoprostanes.

### 4.14. Statistical Analysis

We conducted two-tailed Student’s *t*-tests, one-sample Student’s *t*-tests, and two-/three-way ANOVA tests (Tukey’s post hoc tests) to analyze data. In cases where normality and/or homoscedasticity assumptions were not met, Mann–Whitney tests were employed. A *p*-value of 0.05 or less was considered significant. Data are shown as the mean ± SEM or as the median ± interquartile range (IQR). GraphPad Prism 9.1.0 software (La Jolla, CA, USA) or SPSS Statistics Version 23.0 (for Windows) was employed to run statistical analyses.

## 5. Conclusions

The present study demonstrates that consuming an HFD with an elevated omega-6-to-omega-3 ratio as compared to the CD (25:1 vs. 8.4:1) does not aggravate the outcome of AD regardless of the stage of amyloid pathology, suggesting that many neurobiological processes relevant for AD are not directly dependent of the PUFA intake. This report is consistent with the new view of nutrition experts who have recognized the paradoxical effect of a high-fat diet and have modified their dietary recommendations accordingly.

## Data Availability

The raw data supporting the conclusions of this article will be made available by the authors, without undue reservation, upon reasonable request.

## Supplementary Materials

The following supporting information can be downloaded at: https://www.mdpi.com/article/10.3390/ijms242317009/s1.
