# Supplementary material for: The Effect of Fat Intake with Increased Omega-6-to-Omega-3 Polyunsaturated Fatty Acid Ratio in Animal Models of Early and Late Alzheimer’s Disease-like Pathogenesis
Source: Int J Mol Sci. 2023 Nov 30;24(23):17009. doi: 10.3390/ijms242317009 (PMC10707298; doi:10.3390/ijms242317009)
Supplement: Supplementary file 1 [file ijms-24-17009-s001.zip › ijms-2685290-supplementary.pdf]

Supplementary Information

Table S1. Levels of triglycerides, glucose, total cholesterol, high-density lipoprotein (HDL) and low-density lipoprotein (LDL) in fasted WT and Tg-McGill rats fed with control diet (CD) or high-fat diet (HFD)

Experimental groups		Triglycerides (mg/dL)	Glucose (mg/dL)	Total cholesterol (mg/dL)	HDL (mg/dL)	LDL (mg/dL)
Rats	**WT-CD**	105.0 ± 7.1	102.4 ± 1.99	64.4 ± 4.98	22.1 ± 1.77	21.3 ± 3.01
	**WT-HFD**	144.6 ± 26.4	109.8 ± 1.48	56.9 ± 5.73	22.8 ± 2.22	12.6 ± 2.75
	**Tg-McGill-CD**	75.3 ± 7.1	98.90 ± 1.81	32.5 ± 3.45	14.0 ± 1.22	6.6. ± 1.95
	**Tg-McGill-HFD**	183.8 ± 76.6	103.5 ± 1.45	44.3 ± 9.08	19.0 ± 2.04	5.0 ± 4.20

Fasted plasma from rats was collected at the end of the study and levels of triglycerides, glucose, total cholesterol, HDL and LDL levels were determined. All parameters were found to be within the expected physiological ranges in all of the groups. Values are expressed as the mean ± SEM. N=15-11 rats/group. WT-CD, wild-type rats fed with a control diet (CD); WT-HFD, wild-type rats fed with a high-fat diet (HFD); Tg-McGill-CD, McGill-R-Thy1-APP transgenic rats fed with a CD; Tg-McGill-HFD, McGill-R-Thy1-APP transgenic rats fed with a HFD; HDL, high-density lipoproteins; LDL, low-density lipoproteins.

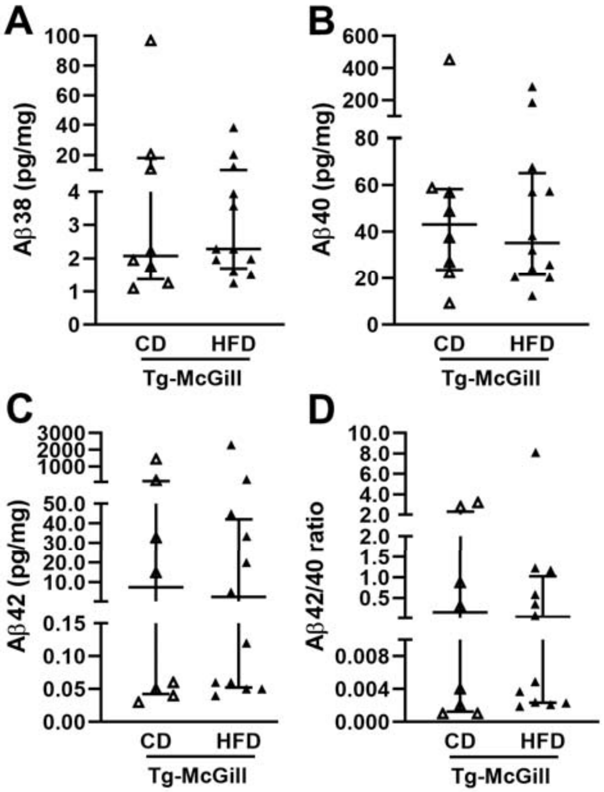

Figure. S1. Hippocampal concentration levels of Aβ isoforms and Aβ42/40 ratios in Tg-McGill rats fed with a control diet (CD) or a high-fat diet (HFD). Non-parametric Mann-Whitney tests were performed to compared hippocampal concentration levels of **(A)** Aβ38 (U=43.5, p=n.s.), **(B)** Aβ40 (U=47.0, p=n.s.), **(C)** Aβ42 (U=42.5, p=n.s.) and **(D)** Aβ42/40 ratios (U=44, p=n.s.) between Tg-McGill-CD and Tg-McGill-HFD rats. These results indicate that significant differences were found for neither any of Aβ isoforms nor for Aβ42/40 ratios. Values are expressed as the median and interquartile range (IQR) with individual values superimposed. N=8-12 rats per group. Tg-McGill, McGill-R-Thy1-APP transgenic rats; CD, control diet; HFD, high-fat diet.

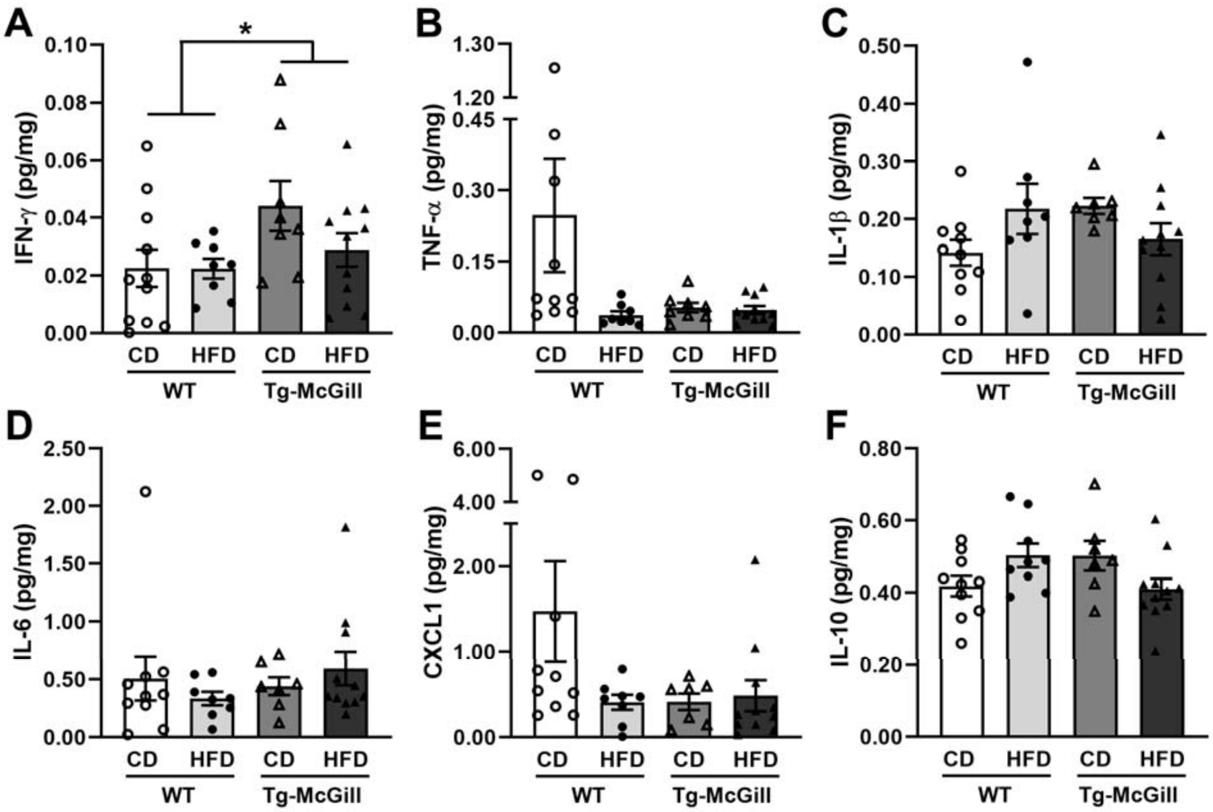

Figure S2. Hippocampal cytokine and chemokine concentration levels of WT and Tg-McGill rats exposed to a control diet (CD) or a high-fat diet (HFD). For each cytokine and chemokine, a two-way ANOVA test was performed with genotype (WT vs. Tg-McGill) and diet (CD vs. HFD) as between-subject factors. In none of the cases the interaction genotype x diet was significant, so only main effects are reported if they were significant. Plasma concentration levels of **(A)** IFN-γ (genotype factor: $F=4.72$, $^{*}p=0.037$); **(B)** TNF-α; **(C)** IL-1β; **(D)** IL-6; **(E)** CXCL1; **(F)** IL-10. These results indicate that Tg-McGill rats show significantly increased brain levels of IFN-γ compared with WT rats, regardless of the type of diet. Data are shown as the mean ± SEM with individual values superimposed. N=7-11 rats per group. WT, wild-type rats; Tg-McGill, McGill-R-Thy1-APP transgenic rats; CD, control diet; HFD, high-fat diet.

Table S2. Expression of nitroxidative stress parameters in hippocampal samples from transgenic rats

Experimental groups		Parameters of nitroxidative stress		
		NO_2-Tyr (µmol/mol Tyr)	ADMA (µmol/mg)	GS (U/mg)
Rats	WT-CD	26.9 ± 3.3	0.40 ± 0.11	0.60 ± 0.05
	WT-HFD	33.6 ± 0.6	0.36 ± 0.07	0.55 ± 0.08
	Tg-McGill-CD	26.6 ± 7.4	0.51 ± 0.07	0.42 ± 0.01
	Tg-McGill-HFD	39.2 ± 5.4	0.34 ± 0.04	0.46 ± 0.14

Non-significant differences were detected between groups for any of the parameters of nitroxidative stress (two-way ANOVA tests with genotype and diet as between-subject factors were performed). Nevertheless, a non-statistical trend was observed in Tg-McGill-HFD rats that showed higher hippocampal values of NO_2-Tyr than Tg-McGill-CD. Values are expressed as the mean ± SEM. N=3-5 rats/group for NO_2-Tyr; N=3-4 rats/group for ADMA and GS activity. NO_2-Tyr, protein nitration; ADMA, asymmetric dimethylarginine; GS, glutamine synthetase; WT-CD, wild-type rats fed with a control diet (CD); WT-HFD, wild-type rats fed with a high-fat diet (HFD); Tg-McGill-CD, McGill-R-Thy1-APP transgenic rats fed with a CD; Tg-McGill-HFD, McGill-R-Thy1-APP transgenic rats fed with a HFD.
